# A literature review: the role of the private sector in the production of nurses in India, Kenya, South Africa and Thailand

**DOI:** 10.1186/1478-4491-11-14

**Published:** 2013-04-12

**Authors:** Jaratdao Reynolds, Thunthita Wisaijohn, Nareerut Pudpong, Nantiya Watthayu, Alex Dalliston, Rapeepong Suphanchaimat, Weerasak Putthasri, Krisada Sawaengdee

**Affiliations:** 1 Faculty of Nursing, Siam University, Bangkok, 10160, Thailand; 2 International Health Policy Program, Ministry of Public Health, Nonthaburi, 11000, Thailand; 3 Faculty of Nursing, Siriraj Hospital, Mahidol University, Bangkok, 10700, Thailand

## Abstract

**Background:**

The demand for nurses is growing and has not yet been met in most developing countries, including India, Kenya, South Africa, and Thailand. Efforts to increase the capacity for production of professional nurses, equitable distribution and better retention have been given high strategic priority. This study examines the supply of, demand for, and policy environment of private nurse production in four selected countries.

**Methods:**

A scoping systematic review was undertaken to assess the evidence for the role of private sector involvement in the production of nurses in India, Kenya, South Africa, and Thailand. An electronic database search was performed, and grey literature was also captured from the websites of Human Resources for Health (HRH)-related organizations and networks. The articles were reviewed and selected according to relevancy.

**Results:**

The review found that despite very different ratios of nurses to population ratios and differing degrees of international migration, there was a nursing shortage in all four countries which were struggling to meet growing demand. All four countries saw the private sector play an increasing role in nurse production. Policy responses varied from modifying regulation and accreditation schemes in Thailand, to easing regulation to speed up nurse production and recruitment in India. There were concerns about the quality of nurses being produced in private institutions.

**Conclusion:**

Strategies must be devised to ensure that private nursing graduates serve public health needs of their populations. There must be policy coherence between producing nurses for export and ensuring sufficient supply to meet domestic needs, in particular in under-served areas. This study points to the need for further research in particular assessing the contributions made by the private sector to nurse production, and to examine the variance in quality of nurses produced.

## Background

In January 2004, a high level forum on the Health Millennium Development Goals (MDGs) reported that there was a need to urgently address the current human resources for health crisis [1]. In response to this crisis, the World Health Organization (WHO) has identified a minimum target threshold for combined doctor, nurse and midwife density of 2.28 per 1000 of the population; below this a health workforce is unlikely to be able to provide sufficient coverage for essential interventions [1]. Fifty-seven countries, mostly in sub-Saharan Africa but also a number of Asian countries, were identified as falling below this threshold [1,2].

In these countries, nurses are recognized as a key component of health care systems fulfilling a wide range of roles, especially where there is a shortage of other health workers [3]. This is particularly the case in primary healthcare. Besides this, nurses are responsive to an increased demand for health services caused by changing demographic [4,5], economic [4,6-8], and epidemiological factors [4,5,9-13]. Effective functioning health systems are thus difficult to achieve if nurses remain scarce [14]. In 2006 the WHO suggested that national governments must anticipate a growing role for the private sector in reducing this problem by increasing the production of nurses [1]. It is clear that nurses will continue to be a key part of health systems but there is a lack of evidence examining the ways in which governments have managed the opening of markets to the private sector, and the contribution that the private production of nurses is making to wider health systems.

This study seeks to examine the supply of, and demand for, private nurse production and the policy environments in which nursing production institutions are operating. It then proceeds to discuss the contributions that private education institutions make to meet health workforce challenges and the risks and opportunities that accompany private involvement in nurse production.

This review was developed as part of the Resilient and Responsive Health Systems (RESYST) project. RESYST conducts collaborative research on health systems, including on the theme of HRH. Partners working on this theme are the African Medical and Research Foundation, Kenya (AMREF), the International Health Policy Programme, Thailand (IHPP), the Indian Institute of Technology Madras (IITM) and the University of Witwatersrand, South Africa. Consequently, India, Kenya, South Africa and Thailand are the four countries that are the focus of this research. The four countries studied represent a broad-ranging demographic, economic and health status. More information on the basic indicators of these four countries is presented in Additional file 1.

## Methods

**Figure 1:**
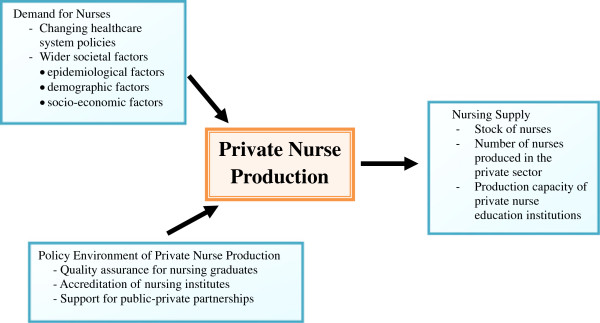
Conceptual framework of the literature review.

A scoping systematic review was conducted to reduce bias and the element of chance. A roundtable discussion amongst researchers was convened to develop the conceptual framework, identify key research questions and determine the subject of the review. Once the information for the research questions was adequately retrieved, all researchers convened another roundtable meeting to analyze the extent of the contribution that private nurse production makes to health systems, and synthesize the knowledge and policy recommendations of this study. Figure 1 provides the framework of the review in line with a number of questions/themes: 1) demand for nurses, 2) nursing supply and 3) policy environment of private nurse production.

Demand for nurses: what was the demand for nurses and its trend in the past decade? How was this affected by changing health needs caused by demographic, epidemiological, and socio-economic changes in the four countries studied, or international demand for nurses from the said countries?

Nursing supply: what was the supply of nurses and its trend over the past decade? What is the extent of nursing supply by the private sector? Supply was defined as the number of nurses overall (stock of nurses), the actual number of nurses produced in the private sector, and the total production capacity of private nurse education institutions, for example, number of schools, or number of teaching staff, regardless of whether or not this capacity is fully utilized.

Policy environment of private nurse production: what, and how, were the policy contexts affecting private nurse production? These included policies to ensure the quality of private nurse graduates, the accreditation of nurse training quality, and the promotion of public-private partnerships in nurse education. The selection of these issues came from brainstorming among the authors, along with consultation with senior officers in the Thailand Ministry of Public Health (MoPH).

Selected articles were collated and appraised with regards to the above questions. All articles were retrieved electronically by one, or other, of two parallel approaches: first, searches from the following electronic literature databases, namely, PubMed, Science Direct Journal of Professional Nursing, Google Scholar, BioMed Central (BMC) Human Resources for Health journal, BMC Medical Education journal, and BMC Nursing’ journal; second, purposive searches from the websites of HRH-related organizations and networks such as, the Ministry of Health (MoH), WHO, Global Health Workforce Alliance (GHWA), World Bank (WB), International Council of Nurses (ICN), the Asia Pacific Action Alliance on Human Resources for Health (AAAH).

The list of key words applied in Google Scholar, BMC and Science Direct was: nurses, ‘private’, ‘production’, ‘supply’, ‘demand’, ‘employment’, ‘responsiveness’, ‘migration’, ‘quality’ and ‘equity’. The keywords used in PubMed corresponded to, but were modified from, those applied in other search engines in order to fit the medical subject headings (MeSH) terms. They were ‘Nurses’, ‘Private Sector’, ‘Supply and Distribution’, ‘Health Services Needs and Demand’, ‘Employment’ and ‘Emigration and Immigration’. Due to time and resource constraints, keywords beyond these MeSH terms were not applied. This resulted in a pool of 657448 references.

Since this study focuses primarily on the four RESYST countries, namely, India, Kenya, South Africa and Thailand, these countries’ names were also applied in every search engine in accordance with the Boolean search strategy (‘India’ OR “Kenya” OR “South Africa” OR “Thailand”). To focus the search, the Boolean search strategy was further utilized. The word “Nurses” was combined with “(“India” OR “Kenya” OR “South Africa” OR “Thailand”)” and one of the other key terms, either “private”, “production”, “supply”, “demand”, etcetera. Language limitations were also imposed: only articles published in English were retrieved. Other limitations were, ‘human not animal’ and ‘published between January, 1st, 2002 and December, 31st, 2011’.

Most articles were retrieved from PubMed and Google Scholar. After combing key terms from the search strategy, 463 potentially relevant articles were selected. Duplicate data were excluded, which left a total of 206 articles for further review. The software EndNote Version X4 was used to store and track the search results from electronic literature databases in a computerized and retrievable format. Once the articles were retrieved, they were entered into the selection process. The first stage of the selection process saw all article abstracts and titles assessed by two reviewers (JR and RS). Only 19 articles relevant to the above conceptual frame work and with fully accessible texts underwent the second stage of the selection process.

The separate purposive search from pertinent agency websites yielded 56 articles. Of these, four were excluded on the basis of duplication. After being reviewed by two independent reviewers, 52 articles were considered potentially relevant; full articles were retrieved, read and discussed by two reviewers (JR and RS): at this stage only 27 articles matching with the research questions were identified. Combined with the 19 articles retrieved from the systematic search, this resulted in a total of 46 papers for assessment in the roundtable discussion with all authors. The result of this discussion saw 23 papers excluded, leaving a total of 23 articles.

**Figure 2:**
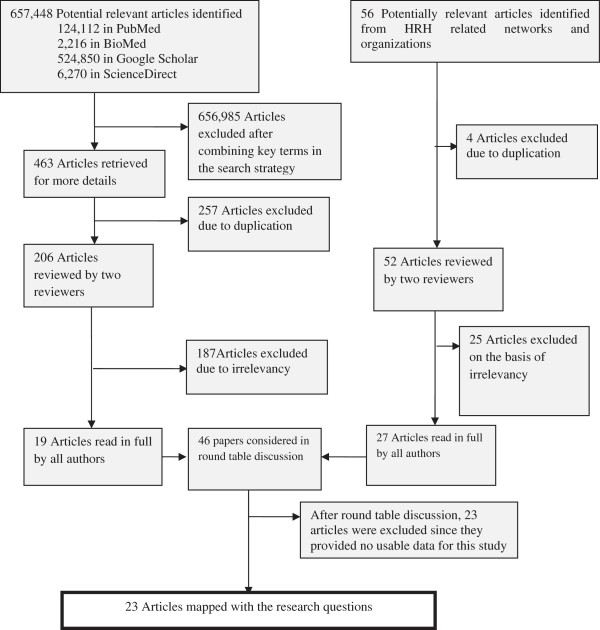
Flow chart of the study selection process.

The flow of the article selection process is summarized in Figure 2, and Table 1 provides detailed results of the systematic search.

**Table 1:** Search strategy and result

**Databases**				
**Keywords**	**PubMed**^**1**^	**Science Direct**	**Google Scholar**^**2**^	**Biomed**^**3**^
1. Nurses^4^	18917	2031	22500	365
2. Private/“Private Sector”	2852	475	29600	284
3. Production	NA	181	134000	92
4. Supply/“Supply and distribution”	15764	315	33200	176
5. Demand/“Health Services Needs and Demand”	19989	753	27300	215
6. Employment	18726	488	26700	197
7. Responsiveness	NA	71	9250	35
8. Migration/“Emigration and Immigration”	5893	78	43300	107
9. Quality	NA	1615	150000	645
10. Equity	NA	64	20300	83
11. “India” OR “Kenya” OR “South Africa” OR “Thailand”	41971	199	28700	17
Total potential articles from all search engines = 657448				
1. AND 11.	176	168	213	12
2. AND 12.	1	58	7	9
3. AND 12.	NA	11	0	3
4. AND 12.	26	26	2	4
5. AND 12.	16	55	0	7
6. AND 12.	11	50	0	6
7. AND 12.	NA	6	0	0
8. AND 12.	6	21	5	6
9. AND 12.	NA	110	1	9
10. AND 12.	NA	4	0	3
Total potential articles from keywords 12 to 21 after applying Boolean search strategy = 463				
After excluding 257 duplicate articles, only 206 articles entered the selection process				

^1^In PubMed, only the Keywords after ‘/’ (or the MeSH terms) were applied. ^2^Confined to articles whose title contained the selected keywords. ^3^Confined to articles whose title, or abstract, or text, contained the keywords except for keyword number 11 (country names), which was required to be present in the title. ^4^This word was applied in all search engines since it exactly matched the MeSH term. NA, not applicable.

## Findings

Contents of the final 23 articles were mapped with the research questions: demand for nurses, supply of nurses (stock of nurses, number of nurses produced in the private sector, and production capacity of private nurse institutions), and policy environment of private nurse production, see Table 2 below.

**Table 2:** Mapping research questions with selected articles

**Author**	**Year**	**Demand for nurses**	**Supply**			**Policy environment of private nurse production**
			**Stock of nurses**	**Number of nurses produced in private sector**	**Production capacity of private nurse institutions**	
The Kenya Health Workforce Project [24]	2012				35 (51%) of total 68 nursing institutions were privately run.	
					More staff in private institutions than public institutions (tutor-student ratio 1:14 in private and 1:40 in public)	
Kanchanachitra C, *et al*. [7]	2011	Acceleration of nursing production to achieve MDGs				
Rao M, *et al*. [8]	2011	Economic growth			95% of all nurses produced by private institutions	
		Introduction of UHC				
		Realignment of health system focusing on primary health care			Quality of nurses produced in private sector due to shortage of staff and facilities	
		Increase in NCD prevalence				
Ndumbe NP [10]	2011	To serve primary health care				
		To achieve MDGs				
		To reach minimum acceptable population coverage				
Rao DT [15]	2011	Towards UHC				
		Focusing on primary health care				
		Increase in NCD prevalence				
		To achieve MDGs				
The Asia Pacific Action Alliance on Human Resources for Health (AAAH) [22]	2011		Maldistribution - density in Bangkok 5 times higher than the rest of the country	Thailand - new graduates from private sector - increase of 24.1% between 2006 and 2010		India - nursing council regulates facilitated scaling up of nurse production.
Bangdiwala S, *et al*. [16]	2010	To serve primary health care				
		To achieve MDGs				
Wibulpolprasert S, *et al*. [23]	2010				10 (14%) out of 64 nursing schools were privately run.	
Gross JM, *et al*. [12]	2010	To serve primary health care				Kenya - national plan to speed up hiring new nurses and utilizing public-private partnership
		To achieve MDGs				
Pagaiya N and Noree T [4]	2009	Changing demographics, economics and epidemiology				
		Towards UHC				
		To serve primary health care				
		Expansion of private provision due to medical hub policy				
George G, *et al*. [20]	2009		Maldistribution and shortage in underserved area			
Krupp K and Madhivanan P [17]	2009	To achieve MDGs				
Matsuno A [27]	2009					Thailand - private institutions produced nurses for their own hospitals.
Adano U [13]	2008	Epidemiological changes				
		Increase in public health care services				
Wadee H and Khan F [21]	2007		Maldistribution and shortage of nurses			
Connell J, *et al*. [11]	2007	Demographic changes				
WHO [5]	2007	Demographic and epidemiological changes				
Khadria B [18]	2007	Increasing international outward migration				India, support of working abroad
						India, state government facilitates export market
Kirigia JM, *et al*. [19]	2006	International brain drain				
		to achieve MDGs				
Subedar H [9]	2005	Epidemiological factors	Maldistribution and shortage of nurses	South Africa - private sector produced 66.3% of enrolled nurses in 2004		
Academy for Nursing Studies, Hyderabad [6]	2005	Economic growth				
		Increase in primary health care services				
		Epidemiological changes				
Jindawatana A, *et al*. [25]	1998				Lower quality of private graduation	Mandatory rural service
						Quality assurance and accreditation to oversee both public and private production
Chunharas S, *et a*l. [26]	1997					Efficient management through stakeholder interface
						Mandatory rural service

MDG, millennium development goal; UHC, universal health coverage; NCD, non-communicable chronic disease.

### Demand for nurses

The studies examined show that the factors affecting the demand for nurses are complex and can be divided into two main categories, namely, changing healthcare system policies, and wider societal factors. Changing healthcare systemic policies which have increased the demand for nurses in all four chosen countries have included the introduction of new financing or insurance systems, and the introduction of universal healthcare coverage (UHC) in some countries [4,8,10,15]. Other policy issues have included the realignment of healthcare systems towards a focus on primary healthcare [4,8-10,12,16], and acceleration to achieve the health MDGs [7,10,12,15-17]. In Thailand there has also been the additional issue of the expansion of private provision of healthcare with the introduction of policies promoting Thailand as a medical hub [4,7].

Wider societal factors at both the domestic and global levels, have also led to an increasing demand for nurses in all four chosen countries: these have included ageing populations [4,11] and increased prevalence of non-communicable chronic diseases (NCD) [4,5,9,10,12,13], the spread of new and re-emerging diseases [4,5], increased public demand for healthcare services [8,13], and socio-economic fluctuations [4,6-8]. There are also numerous cases of a mismatch of demand and supply leading to localized shortages, especially in rural or hardship areas [5]. As well as domestic demand these factors result in changing global demand for nurses, causing international migration. This is a particular problem for Kenya and India where nursing supply is still in crisis [18,19].

### Nursing supply

Table 3 displays the density of nurses (the ratios of nurses to 1000 in the population) in the four selected countries, between 2000 and 2010. In South Africa, nurse density was 4.08 per 1000; meanwhile, Kenya had a significantly lower density of 1.18 nurses per 1000. This level was only marginally exceeded by India, which had a density of 1.30 nurses to 1000 and likewise, Thailand, which had a density of 1.52 nurses to 1000.

**Table 3:** Nursing and midwifery density for the period 2000 to 2010

**Country**	**Number of nurses and midwives**	**Density**
		**(per 1000 population)**
India	1430555	1.30
Kenya	37113	1.18
South Africa	184459	4.08
Thailand	96704	1.52

Source: World Health Statistics, WHO, 2011.

While the overall density of nurses was significantly higher than in the other three countries, South Africa still suffered from the maldistribution of nurses, shortages in under-served and rural areas, and still had insufficient nurses to meet public health needs [9,20,21]. Maldistribution was also a significant problem in Thailand where the density of Thai nurses working in the capital Bangkok was more than five times higher than that of the rest of the country [22].

### Number of nurses produced in private sector

In 2004 South Africa produced 35266 enrolled nurses, who had completed a two-year training programme [9]. The number of enrolled nurses produced by the private sector has rapidly overtaken the number produced by the public sector. In 2001 the private sector produced 45.2% of enrolled nurses, but by 2004 this had increased to 66.3% [9]. This is not the same in other cadres; in 2004 South Africa had 98,490 professional nurses who completed a four-year degree-level qualification. The literature review provided no evidence of private sector involvement in production of this cadre in South Africa. In Thailand, all nurses are professional nurses, who undertake a four-year programme. Of all new nursing graduates 19.6% came from private nursing education institutions in 2006 and by 2010 this figure had increased to 24.1% [22]. This shows the growing importance of the private sector in nurse production in Thailand. The reviewed papers did not have evidence of the overall number of nurses produced in the private sector in either India or Kenya.

### Production capacity of private nurse education institutions

The literature review found no evidence of the number of staff, the financing, or details of the facilities available in private nursing education institutes in any of the four selected countries. However, there was sufficient evidence of the overall number of teaching institutions. In India 88% of all nursing education institutions were in the private sector, which was responsible for producing 95% of all nurses [8,22]. Of these, only 9% of all nursing schools are located in states experiencing nurse shortages [8]. In 2009 Thailand had 74 nursing schools, 64 (86%) of which were public, and 10 (14%) were private institutions [23]. In Kenya the private sector has a large role with 35 (51%) of the total number of 68 nursing institutions being privately run [24].

The quality of nurses produced in these private institutions has been questioned. In India it was reported that approximately 61% of nursing colleges in the country were unsuitable for training nurses due to an acute shortage of facilities and faculties [8]. In Thailand it has been noted that nursing graduates from private institutions have been of a lower quality than those graduating from public institutions. It has been claimed that this may result from private institutions accepting students of lower quality than those accepted to study in public institutions [25]. In Kenya private institutions appear to have a higher ratio of tutors to students, with one tutor to fourteen students in private institutions compared to one tutor to forty students in public institutions [24]. The reviewed papers did not provide information of the production capacity of private nurse education institutions in South Africa.

### Policy environment of private nurse production

At the institutional level in Thailand both public and private institutions employ financial support schemes to subsidize students through soft education loans. These are repayable through either customized repayment schemes, or through serving a period of work in rural public healthcare services [25,26]. Despite the efforts that have been made, there are concerns about the efficiency, quality and effectiveness of the mechanisms and regulation of private sector nurse production [25].

At the national level, Thailand has established efficient quality assurance and accreditation systems to oversee both public and private institutions in nurse production, as well as introduction of a requirement for all public and private graduates to pass a national licensing examination [25] organized by the Thailand Nursing and Midwifery Council. Re-licensing every five years is a mandatory requirement achieved through earning credits from the in-service continued nursing education.

In Kenya the implementation of a national Emergency Hiring Plan has involved upgrading training to speed up the hiring of new nurses, utilizing public-private partnerships [12]. In India nursing councils have modified the regulations around establishing new nursing schools and colleges, which has facilitated the scaling up of nurse production [22] in the face of rapidly increasing outward migration of nurses [18].

In response to the advent of the Association of Southeast Asia Nations (ASEAN) Economic Community in 2015, Thailand has chosen to further medical-hub policies that seek to attract a large number of international patients seeking medical care in Thailand. This has created a new market where Thai nurses can find better employment in private hospitals within the country rather than seeking outward migration. This has generated an increase in the number of private nursing schools that provide bilingual English-Thai programmes, most of which aim to use the nurses that graduate from their courses within their own hospitals [27]. The reviewed articles did not show concrete evidence of the policy environment regarding private nurse production in South Africa.

## Discussion

The articles in this review demonstrate several common findings vis-à-vis supply of, and demand for, nurse production in the private sector; however it should be noted that this review is subject to a number of limitations that are set out separately below. What is clear is that in all the selected countries the demand for nurses is rising. This demand has not yet been met, despite the fact that the supply of nurses has been increasing significantly, particularly from private institutions. While this was universal in the four countries reviewed, the policy environments affecting the operation of private nurse education institutes, varied from country to country. The following section will analyze some key concerns, and draw lessons from the broader experience of countries other than those selected in this study.

### Outputs from private nursing education institutions

In Thailand, it was argued that private education institutes would produce greater efficiency in education management because they are commonly more flexible, competitive and efficient than those run under a central bureaucratic system [25]. This flexibility has been seen in the Philippines where in periods of high demand private institutes have been able to rapidly scale up the production of nurses. Between 2005 and 2007 private institutes produced approximately 55000 nurses per year, a seven-fold increase on the numbers produced in the years 2000 to 2004 [7]. These private institutes have consciously focused on producing nurses for the international labour market, and this has successfully generated employment and remittances of foreign exchange for a country that has been in severe need of both [7,19,28].

However this rapid scaling up has come at the cost of a reduction in the quality of graduates. In the period of intensive increase in production identified above (2005 to 2007), fewer than 50% of graduates passed licensing examinations [7]. While the number of successful graduates still represented a three and a half-fold increase on the annual rate of production of the preceding five years, it shows that flexibility comes at a price.

It is important to question the extent to which private nursing education institutions can fit into wider planning of national nurse production. For example, in South Africa the private sector has produced only enrolled nurses while it is professional nurses who make up the bulk of South Africa’s nurse supply [9].

A risk accompanying rapid expansion of nursing production is the difficulty of finding suitable teachers, and the excessive utilization of existing teachers, as has been reported in India [8]. That said, while high student-to-tutor ratios are a concern, quality is multi-faceted, so a high tutor-to-student ratio alone is no guarantee of quality education. A further potential risk of private production is that the tuition fees are likely to be higher than in public institutions due to a lack of state subsidies. This may result in the inequitable recruitment of students, with those students from wealthier, and especially urbanized, backgrounds being unfairly advantaged [25].

There is no strong evidence that private nursing education institutions imbue the same sense of publicservice as state institutions. The decision of nurse graduates from private nursing institutes in India to work abroad, despite a serious shortage of nurses within the country [18], could be interpreted as indicating a lack of commitment to domestic public services. However, this migration appeared to be promoted by government administrators, as much as private nursing teachers. None of the papers studied explored the issues of motivation, intended career path and attitude to rural service of nurses graduating from the private sector. Although beyond the scope of this study, this topic is the subject of debate in many countries and may be an interesting area for future research.

### Utilization of nurses graduating from private nursing education institutions

While bringing the potential to expand nurse production, the involvement of private education institutions may also bring serious risks. In the Philippines, producing an excess of nurses specifically for export has resulted in financial benefits, however, it has also resulted in a worsening skill mix in hospitals within the country [7]. A significant number of experienced nurses have left to work overseas, sometimes resulting in ward or hospital closures. Before migrating to work internationally, nurses in the Philippines need to gain work experience. This shortage of senior nurses to supervise new graduates results in a high number of newly registered nurses experiencing bottlenecks; they are unable to gain experience to meet the requirements for overseas work and under-qualified to fill domestic vacancies [7].

In India there are shortages of nurses of all kinds, but the presence of private sector agencies, which generate very large profits from training nurses to send overseas, has led to the development of a large export market, and now some state governments are facilitating this exodus. For instance, after investing between US$4700 and US$ 7000 USD in training each nurse, Indian companies can make US$ 47000 for each nurse placed abroad [18]. The incentives provided by the large profits generated by the schools and agencies which produce nurses for export appear to be an overriding concern for the domestic supply of nurses [18].

In Kenya, the export of nursing staff has resulted in an impoverishment of the health sector and has not resulted in the high level of remittances seen in the Philippines. The comparatively higher wages and fees associated with supplying nurses to the Organisation for Economic Cooperation and Development (OECD) and Middle-Eastern countries when compared to domestic markets mean that private institutions producing nurses for profit are naturally drawn to this wider market [19].

### Policy considerations

The entrance of private interests to nurse production has arisen as a result of governments’ loosening of legislation to intentionally allow private production of health professionals in some countries as has been the case in Thailand, and a lack of legislation or other regulatory instruments in other countries, including India [8,25]. A number of strategies must be comprehensively devised to ensure that those nurse graduates serve the health needs of their populations. There must be a balance struck between producing nurses for export, and ensuring equitable distribution, and sufficient supply and skill-mix for domestic markets [18,19]. Long-term human resource planning and effective government oversight is also required. This should include policies to ensure the quality of nurse graduates, and upgrade the capacity of teaching staff in nursing production institutes [24].

Strategies must be sought, or further improved, in order to cope with the problem of limited national resources, for example, improving management efficiency through stakeholder interface in both public and private nursing schools [26]. Strengthening public-private partnerships is also a potentially valuable strategy that could increase the overall quantity and quality of nurses in response to each country’s need [3,9,21].

### Availability of evidence

While a large number of potential articles were identified for this study, they failed to provide the evidence required to answer a number of the questions raised in this paper. This indicates that there is a great deal more research required in this area. Areas of particular weakness include the following: 1) lack of clear statistical information about the production of nurses in the private sector; 2) lack of information regarding the number of teaching staff, the financing and facilities of the private nurse education institutions, and 3) inadequate information about the employment of private nursing graduates, for example, in urban versus rural, public or private health sector, or employment abroad.

This study found insufficient evidence to discern the full extent to which private nursing education contributes to current health systems. More primary research is required in evaluating the following areas: equity in student recruitment, assessment of students and graduates in terms of quality and productivity, and responsiveness to health system needs.

### Limitations of the study

Despite a concerted attempt to conduct an extensive review, this study contains a number of limitations. The requirement that the selected articles be written only in English may have excluded useful data published in other languages. It is also of concern that the mechanism employed to ensure the quality of papers assessed may not have been sufficiently stringent. The systematic search strategy employed in this study was also confined to a limited number of search engines. Additional databases including those related to social sciences and humanities were not searched. One critical limitation is the heterogeneity of the types and quality of the articles retrieved. Most of the selected articles were reports or reviews of secondary data, and there was little evidence from well-designed primary studies. The limited amount of material available also meant that in many areas, the triangulation of data was not possible.

## Conclusion

It should be noted that changes in the labour market, regulatory environment and the demand for nursing personnel at domestic and international levels significantly determine the direction and the operation of private nursing education institutions. While the private sector can provide flexibility in production, governments should ensure that nursing graduates from public and private education institutions are of sufficient quality and meet the health needs of their populations. This can be achieved through effective standardized accreditation and licensing systems. Countries that are experiencing nursing shortages, while seeking to encourage increased private production of nurses, should also make meeting domestic need the first concern, before then considering serving the international market. There is an evident need for further primary research to ascertain the exact nature of the contribution of the private sector to nurse production, and to examine the variance in the quality of nurses produced.

## Abbreviations

AAAH: Asia Pacific Action Alliance on Human Resources for Health; AMREF: African Medical and Research Foundation; ASEAN: Association of Southeast Asia Nations; GHWA: Global Health Workforce Alliance; HRH: Human Resources for Health; ICN: International Council of Nurses; IHPP: International Health Policy Programme; IITM: Indian Institute of Technology Madras; MDG: Millennium development goal; MeSH: Medical subject headings; MoH: Ministry of Health; MoPH: Ministry of Public Health; OECD: Organisation for Economic Cooperation and Development; RESYST: Resilient and Responsive Health Systems; UHC: Universal healthcare coverage; WB: World Bank; WHO: World Health Organization

## Competing interest

The authors declare that they have no competing interest.

## Authors’ contributions

JR, KS, WP and RP jointly designed the study; Data collection was conducted by JR and RP. JR, RP, NW, NP, TW, and AD contributed to analysing the data. All authors contributed to the drafting, revision, finalization and approval of the manuscript.

## Supplementary Material

Additional file 1 Basic indicators of the four selected countries. Click here for file

## References

[B1] WHO The world health report 2006: working together for health 2006 Geneva: World Health Organization

[B2] Anyangwe SC Mtonga C Inequities in the global health workforce: the greatest impediment to health in sub-Saharan Africa Int J Environ Res Public Health 2007 4 2 93 100 10.3390/ijerph2007040002 17617671 PMC3728573

[B3] Wildschut A Mqolozana T Shortage of nurses in South Africa: Relative or absolute. Case study report compiled for the DoL study: A multiple source identification and verification of scarce and critical skills in the South African labour market 2008 Department of Labour South Africa, HSRC/DoL To be made available on CD with Erasmus and Breier

[B4] Pagaiya N Noree T Bank W Thailand’s Health Workforce: A Review of Challenges and Experiences 2008 World Bank: Washington

[B5] WHO Scaling up health workforce production: a concept paper towards the implementation of World Health Assembly resolution WHA 59.23 2007 Geneva: World Health Organization

[B6] Academy for Nursing Studies H Situation Analysis of Public Health Nursing Personnel in India 2005 New Delhi: Training Division, Ministry of Health and Family Welfare, Government of India

[B7] Kanchanachitra C Lindelow M Johnston T Hanvoravongchai P Lorenzo FM Huong NL Wilopo SA Human resources for health in southeast Asia: shortages, distributional challenges, and international trade in health services Lancet 2011 377 769 781 10.1016/S0140-6736(10)62035-1 21269674

[B8] Rao M Rao KD Kumar A Chatterjee M Sundararaman T Human resources for health in India Lancet 2011 377 587 598 10.1016/S0140-6736(10)61888-0 21227499

[B9] Subedar H Gray A Govender M Gengiah T Singh J The nursing profession: production of nurses and proposed scope of practice South African Health Review 2005 Cape Town: Health System Trust 88 101

[B10] Ndumbe PM The training of human resources for health in Africa Joint Learning Initiative on Human Resources for Health, Rockefeller Foundation, Africa Working Group 2004 Cameroon: University of Yaounde

[B11] Connell J Zurn P Stilwell B Awases M Braichet JM Sub-Saharan Africa: beyond the health worker migration crisis? Soc Sci Med 2007 64 1876 1891 10.1016/j.socscimed.2006.12.013 17316943

[B12] Gross JM Riley PL Kiriinya R Rakuom C Willy R Kamenju A Oywer E Wambua D Waudo A Rogers MF The impact of an emergency hiring plan on the shortage and distribution of nurses in Kenya: the importance of information systems WHO Bulletin 2010 88 824 830 10.2471/BLT.09.072678 PMC2971507 21076563

[B13] Adano U The health worker recruitment and deployment process in Kenya: an emergency hiring program Hum Resour Heal 2008 6 19 10.1186/1478-4491-6-19 PMC2557010 18796157

[B14] Anand S Brnighausen T Human resources and health outcomes: cross-country econometric study Lancet 2004 364 1603 1609 10.1016/S0140-6736(04)17313-3 15519630

[B15] Rao DT Human Resources for Universal Health Coverage, A Case Study of India 2011 New Delhi: Public Health Foundation of India

[B16] Bangdiwala SI Fonn S Okoye O Tollman S Workforce resources for health in developing countries Public Health Rev 2010 32 296 318 10.1093/pubmed/fdq059

[B17] Krupp K Madhivanan P Leveraging human capital to reduce maternal mortality in India: enhanced public health system or public-private partnership? Hum Resour Heal 2009 7 18 10.1186/1478-4491-7-18 PMC2662781 19250542

[B18] Khadria B International nurse recruitment in India Health Serv Res 2007 42 3 Pt 2 1429 1436 17489924 10.1111/j.1475-6773.2007.00718.x PMC1955375

[B19] Kirigia JM Gbary AR Muthuri LK Nyoni J Seddoh A The cost of health professionals’ brain drain in Kenya BMC Health Serv Res 2006 6 89 10.1186/1472-6963-6-89 16846492 PMC1538589

[B20] George G Quinlan T Reardon C Human Resources for Health: A Needs and Gaps Analysis of HRH in South Africa 2009 Durban, South Africa: Health Economics and HIV & AIDS Research Division (HEARD) University of KwaZulu-Natal

[B21] Wadee H Khan F Human resources for health: health care delivery South African Health Review 2007 Durban: Health System Trust 141

[B22] AAAH The 6th Asia Pacific Action Alliance on Human Resources for Health Annual Conference, Capacity Building for HRH Management and Development to Support Universal Health Coverage 2011 Cebu: AAAH

[B23] Wibulpolprasert S Sirilak S Ekachampaka P Wattanamano N Thailand Health Profile 2008–2010 2011 Thailand: Ministry of Public Health

[B24] UNFPA Kenya’s Health Workforce Training Capacity: A Situation Analysis 2010 Kenya Health Workforce Project

[B25] Jindawatthana A Milintangkul U Rajataramya B Future policy options for HRH production in the ministry of public health, Thailand Hum Resour Health Dev J 1998 3 43 54

[B26] Chunharas S Tangcharoensathien V Kittidilokkul S The role of public and private sector in manpower production: a debate Hum Resour Health Dev J 1997 1 77 98

[B27] Matsuno A Nurse migration: The Asian perspective. ILO/EU Asian Programme on the Governance of Labour Migration Technical Note 2009 International Labour Organization

[B28] Alonso-Garbayo A Maben J Internationally recruited nurses from India and the Philippines in the United Kingdom: the decision to emigrate Hum Resour Heal 2009 7 37 10.1186/1478-4491-7-37 PMC2680394 19393080

